# Anterior Cruciate Ligament Reconstruction Surgery: Creating a Permissive Healing Phenotype in Military Personnel and Civilians for Faster Recovery

**DOI:** 10.1093/milmed/usac093

**Published:** 2022-04-07

**Authors:** Jodie L Morris, Peter McEwen, Hayley L Letson, Geoffrey P Dobson

**Affiliations:** Heart and Trauma Research Laboratory, College of Medicine and Dentistry, James Cook University, Townsville 4811, Australia; The Orthopaedic Research Institute of Queensland (ORIQL), Townsville, Queensland, QLD 4812, Australia; Heart and Trauma Research Laboratory, College of Medicine and Dentistry, James Cook University, Townsville 4811, Australia; Heart and Trauma Research Laboratory, College of Medicine and Dentistry, James Cook University, Townsville 4811, Australia

## Abstract

**Introduction:**

Anterior cruciate ligament (ACL) rupture in military personnel and civilians can be a devastating injury. A service member is 10 times more likely to suffer an ACL injury than their civilian counterparts, and despite successful surgical stabilization, 4%-35% will develop arthrofibrosis, over 50% will not return to full active duty, and up to 50% will develop post-traumatic osteoarthritis (PTOA) within 15 years. Equally concerning, woman are 2 to 8 times more likely to experience ACL injuries than men, which represents a major knowledge gap.

**Materials and Methods:**

A comprehensive literature search was performed in December 2021 using structured search terms related to prevalence, risk factors, disease progression, and treatment of ACL injury and reconstruction. The literature search was conducted independently by two researchers using PubMed, Cochrane, and Embase databases, with inclusion of articles with military, civilian, and sex relevance, and exclusion of most papers with a publication date greater than 10 years. The resources used for the review reflect the most current data, knowledge, and recommendations associated with research and clinical findings from reliable international sources.

**Results:**

Currently, there is no effective system-based drug therapy that creates a “permissive environment” to reduce synovial and cartilage stress after ACL injury and reconstruction and prevent secondary complications. We argue that progress in this area has been hampered by researchers and clinicians failing to recognize that (1) an ACL injury is a system’s failure that affects the whole joint, (2) the early molecular events define and perpetuate different injury phenotypes, (3) male and female responses may be different and have a molecular basis, (4) the female phenotype continues to be under-represented in basic and clinical research, and (5) the variable outcomes may be perpetuated by the trauma of surgery itself. The early molecular events after ACL injury are characterized by an overexpression of joint inflammation, immune dysfunction, and trauma-induced synovial stress. We are developing an upstream adenosine, lidocaine, and magnesium therapy to blunt these early molecular events and expedite healing with less arthrofibrosis and early PTOA complications.

**Conclusions:**

ACL injuries continue to be a major concern among military personnel and civilians and represent a significant loss in command readiness and quality of life. The lack of predictability in outcomes after ACL repair or reconstruction underscores the need for new joint protection therapies. The male–female disparity requires urgent investigation.

## INTRODUCTION

Injury to the anterior cruciate ligament (ACL) is one of the most devastating and frequent injuries of the knee.

Kiapour and colleagues^1^ p 20

**FIGURE 1.**
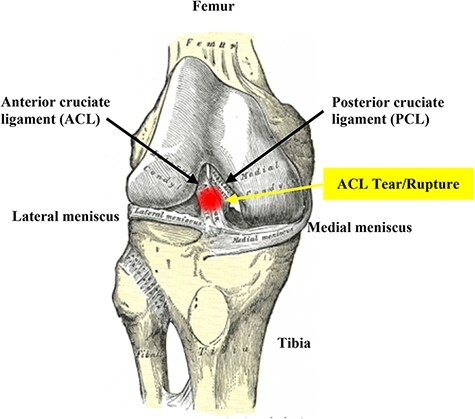
The anterior cruciate ligament is a band of dense connective tissue that prevents the tibia from sliding out in front of the femur as well as provides rotational stability to the knee. The term “cruciate” refers to the crossing over (from “crus” meaning “cross”) of anterior and posterior ligaments. The posterior cruciate ligament (PCL) courses from a more anterior position in the femoral notch to the posterior aspect of the proximal tibia. The medial and lateral collateral ligaments control the sideways motion of your knee and brace it against unusual movement. The average tensile strength for the ACL is 2160 N. This is slightly less than the strength of the posterior cruciate ligament and approximately half as strong as the medial collateral ligament. Mechanoreceptors in the intact ACL contribute toward the functional stability of the knee joint, and injury leads to disturbance of neuromuscular control from their damage or loss. The patella sits in front of the joint to provide protection (not shown).

Anterior cruciate ligament (ACL) rupture is one of the most debilitating musculoskeletal injuries. Despite successful repair and rehabilitation, most people will experience increasing impairment of the joint.^2–4^ The primary function of the ACL is to prevent the tibia from sliding anteriorly past the femur (extended knee) and to stabilize the knee from excessive rotational, pivot-shift movements (Fig. 1). Anterior cruciate ligament injuries are more common than posterior cruciate ligament (PCL) injuries by a factor of ∼ 9:1 largely because the PCL is broader and stronger (Fig. 1).^2,3^ Anterior cruciate ligament injuries range from mild, such as a small tear, to severe when the ligament ruptures completely or separates from the bone^1–3,5,6^ (Table I). Anterior cruciate ligament tears or rupture typically occurs following rapid knee hyperextension, excessive rotational stresses, and/or extreme deceleration forces.^3,7–9^

**TABLE I.** Definitions and Complications after Successful Anterior Cruciate Ligament Surgery

Condition	Definition
Anterior cruciate ligament injury	Injuries range from mild, such as a small tear, to severe, such as when the ligament tears completely or when the ligament and part of the bone separate from the rest of the bone.^3,4,13,20^
Arthrofibrosis (adhesive capsulitis)	Excessive scar tissue formation within the knee joint secondary to an inflammatory process, fibroblast hyperactivity, exaggerated endothelial proliferation, and disorganized deposition of the protein matrix that leads to stiffness, pain and loss of normal motion (loss of flexion, extension or both).^29,67^ Histologically, a dense collagen deposition is common.^20^ Arthrofibrosis can contribute to PTOA, and vice versa.
Post-traumatic osteoarthritis (PTOA)	A form of osteoarthritis from an accelerated form of inflammation, cartilage degeneration and joint dysfunction that causes stiffness and pain.^3,4,31–34^
Surgical trauma	Trauma from surgery itself could add to the molecular events and exacerbate acute joint injury, and later life complications. This area has received little attention.^65^

### Incidence of ACL Tears in Military and General Populations

Anterior cruciate ligament injuries in the U.S. Military service members have a 10-fold higher incidence than that of the general population due to the physically demanding aspects of military duties.

Tennent and Posner^10^ p 119

The incidence of ACL and meniscal tears in the military are up 10 times higher than in the civilian population.^10–13^ This is largely due to the high intensity and frequency of training, frequency of deployment, and other military activities, with the highest rates in the U.S. Army and Special Operations.^13^ Higher rates of ACL injury among military personnel are also linked to higher rates of arthrofibrosis and post-traumatic osteoarthritis (PTOA), which represents a significant loss to command readiness, mental health issues, and loss of quality of life.^13–15^ Of increasing concern, females from military and civilian populations are 2 to 8 times at higher risk for ACL injuries than men^7,14,16,17^ (see below).

In the general population, there are over 2 million ACL injuries each year globally, with an annual growth rate of 4%-6%.^1,5,18^ Over 70% of these injuries are sports-related from football, rugby union, rugby league, gymnastics, netball, basketball, soccer, and skiing.^5,19^ In the United States, ACL tears represent more than 50% of all knee injuries and affect more than 200,000 people each year.^2,20^ European registries report similar results with on average 35 injuries per 100,000 people in Norway, Denmark, Sweden, and Germany.^21^ Higher incidences have been reported in New Zealand and Australia with 58.2 and 77.4 per 100,000 people, respectively.^5,19^ The increasing rate of ACL injuries is skewed toward the younger sporting population (15-24 years), particularly in women.^5,6^ For example, Sutherland and colleagues recently reported a 20% increase in injury rate in females aged between 15 and 19 compared to 10 years ago.^19^ This is an emerging global healthcare problem that needs to be addressed.

### Woman are at Greater Risk of ACL Injuries Than Men

This greater prevalence for ACL injury in young female athletes must be considered to be one of the major problems in sports medicine.

P.A. Renstrom^18^ p1.

The increased risk of ACL injury for females is due to multiple factors including lower limb alignment, intercondylar notch size and shape, joint laxity, hormonal effects, and ligament size.^7,18,22^ Another key risk factor is neuromuscular control of the joint, with females having a reduced electromechanical ability to stiffen the knee joint during rapid movements.^7,23^ Dynamic neuromuscular control requires recruitment of large muscle forces and fast reaction times to generate peak torque to quickly maneuver, land, or change direction.^24^ Women appear to be more “quadriceps dominant,” with lower hamstring recruitment and slower times to peak torque than men.^18^ These differences in neuromuscular control, combined with other factors, may increase a woman’s risk for ACL injury.^24^ However, after 20 years, it is unacceptable that little or no progress has been made to improve prevention measures for ACL injury in women.^17^ In addition to sex-specific differences in the ACL injury rate, the molecular responses to injury and surgical reconstruction may be different between females and males, which is an area requiring urgent investigation. Thus, key to future research is equal male–female representation to understand sex-specific differences after ACL injury and reconstruction.

### Short- and Long-Term Complications of ACL Injury

Outcomes following anterior cruciate ligament (ACL) reconstruction are considered poor. There are many factors which may influence patient outcomes.

Walker and colleagues ^25^ p 1

A major concern following ACL injury is over 50% of military service members are unable to return to full active duty,^10,26^ and a similar figure applies to civilians working in high demand work (49%-63%).^22,27^ Failure to recover full knee function occurs despite receiving the most advanced surgical, rehabilitation, and prevention practices, although treatment of elite athletes have much better outcomes (up to 80% return to their sport).^28^ Notwithstanding a 5%-10% failure rate of ACL reconstruction (ACLR) surgery in the general population,^2,3,20^ reasons for failure to return to preinjury levels fall into four broad categories:^1^ ongoing joint inflammation and scar formation (primary arthrofibrosis),^2^ persistent pain that limits motion,^3^ local infection, and/or^4^ recurrent instability secondary to laxity in the reconstructed ligament^2,3,15,20^ (Table I).

**FIGURE 2.**
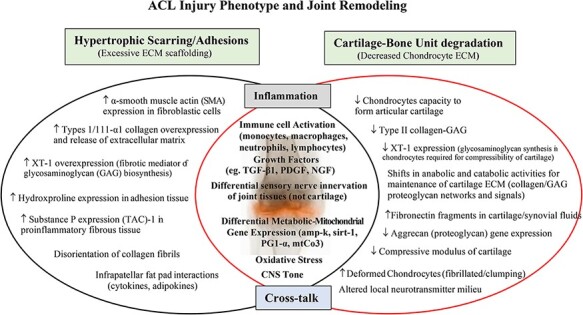
A schematic of the early molecular stages of arthrofibrosis and PTOA within the knee following ACL rupture or tear. Both acute pathologies are driven by inflammation and lead to hypertrophic scarring and cartilage–bone unit degradation. New drug therapies are urgently required to reduce the points of intersection that link early joint inflammation, immune dysfunction, and trauma-induced synovial stress. Restoring balance to cytokine signaling and the synovial expression of anabolic and catabolic genes, and their protein translational pathways, may reduce excessive ECM scaffolding, cartilage degradation, pain, arthrofibrosis, and early PTOA (see text). ACL, anterior cruciate ligament; ECM, extracellular matrix; fibronectin, component of the cartilage matrix; GAG, glycosaminoglycan; Substance P, sensory afferent nerve neurotransmitter that can stimulate cytokine release and fibrogenesis; TAC-1, tachykinin; NGF, nerve growth factor; PTOA, post-traumatic osteoarthritis; XT, anabolic enzyme xylosyltransferase.

Longer-term clinical complications are equally debilitating. Regardless of successful ACL stabilization, 4%-35% of patients will develop progressive arthrofibrosis^29–31^ and roughly 50% of patients will develop PTOA within 15 years^3,4,32–34^ (Table I). Compositional magnetic resonance imaging (MRI) studies suggest that progressive cartilage degeneration begins 1-2 years after the initial injury^4^ and radiographic PTOA indications 10 years after the initial injury.^3,35^ Historically, PTOA onset had been diagnosed by radiographic joint space narrowing; however, the MRI-based studies indicate that the “molecular events” contributing to progressive cartilage degeneration may occur within weeks of the initial ACL injury.^36^ This early, clinically silent, window can be termed “the molecular stage of PTOA,” which may be potentially preventable. Importantly, since PTOA affects the whole joint, arthrofibrosis can contribute to PTOA and vice versa,^34,37^ we need to better understand the cross talk between the two pathologies and design new drugs to restore homeostatic balance (Fig. 2).

### Identifying Current Gaps for Improved Outcomes

ACL reconstruction does not protect articular cartilage from degenerative changes.

Deckers and colleagues ^38^

The lack of predictability in ACL repair or reconstruction outcomes underscores the need for new joint protection therapies. We argue that the development of new therapies requires the recognition of two key features of ACL injury: (1) ACL injury is a system’s failure that affects the whole joint, and (2) the early molecular events define and perpetuate different injury phenotypes.

### ACL Injury is a System’s Failure Affecting the Whole Joint

Anterior cruciate ligament ruptures or tears are rarely isolated injuries since 88% have concomitant structural damage to the knee,^39^ over 50% have lateral or medial cartilaginous menisci tears^9^, 30% have articular cartilage damage,^32,34^ and over 50% have traumatic hemarthrosis or bleeding into peripheral attachments and capsule from injured blood vessels.^39,40^ Hemarthrosis can further decrease cartilage stability and viability.^36^ Thus, the evidence for ACL injury affecting the whole joint is compelling and comes from functional MRI, biochemical, histological, electrophysiological, immunological, metabolic, and biomechanical studies.^2,3,41^ The whole joint is significantly compromised from changes in matrix composition, loss of joint mechanoreceptors, synovial membrane damage, acute swelling, hemorrhage, cellular infiltration into the synovium, inflammatory activation of joint tissue cells, neuromuscular impairment, tendon damage, and bone bruising.^4,34,41^ “The key point is the ACL, and other ligaments, have intimate anatomic and functional relationships to all structures within the knee, and when it is injured the entire knee joint is traumatized.”

### Early Molecular Events Define and Perpetuate Different Injury Phenotypes

The ACL injury phenotype is defined as a proinflammatory, procoagulopathic, proadhesive, prooxidative, profibrotic, procatabolic, and chondral degradative phenotype with neuromuscular and functional deficits.^29,41,42^ Following injury, the joint becomes a chaotic milieu of “damage” signals, which include DNA modifiers, inflammatory amplifiers, injury inducers, degradative enzymes, and cartilage breakdown markers (Fig. 2).^1,42,43^ The type of injury phenotype appears to be dependent on many factors including the severity of ACL injury and extent of trauma to other joint issues, health of the patient, history of pre-existing injury, timing of surgery, sex and age, graft type and positioning, infection status, and postoperative rehabilitation practices.^1,44,45^ Identifying the different early molecular signatures defines the different injury phenotypes.

### Trauma-Induced Immune Activation

Within minutes of injury, the local and systemic immune response is activated.^46,47^ Resident macrophages, natural killer (NK) cells, and fibroblasts from the synovium; lymphocytes, mast cells, and dendritic cells from the perivascular tissues; and osteoclasts from bone marrow are released by local damage stimuli.^46–48^ In the first few hours, these activated cells lead to the influx of blood-borne neutrophils, monocytes, T helper cells, and B cells that enter the joint capsule to facilitate wound healing to initiate cleanup, cell proliferation, and remodeling.^48–51^ M1 macrophages are also activated by complement receptors (C3a, C5a, and C5b), which can induce the activation of the NLRP3 inflammasome to amplify the inflammatory response.^52,53^ Resident innate NK cells also secrete cytokines, such as interferon-γ (IFN-γ) and tumor necrosis factor-alpha (TNF-α), and interact with macrophages, and other immune cells, to enhance the response.^47,54^ These different immune and non-immune cells, through their cytokine networks, play pivotal roles both as activator cells and target effector cells to produce the correct healing response.^46^ Within the joint, the synovial membrane regulates the traffic by maintaining a rich network of sympathetic and sensory nerves, blood vessels, and lymphatic vasculature to promote healing.^48^

### Uncontrolled Inflammation at the Intersection of Early Arthrofibrosis and PTOA

Studies in recent years have unequivocally shown that resolution of inflammation is an actively controlled processes rather than a passive procedure in which the proinflammatory immune cascade in inflammation simply fizzles.

Markus F. Neurath ^55^ p 627

Like the immune system, inflammation is critically important for the normal healing process.^46^ However, when the ACL injury surpasses the body’s normal tolerances, inflammation can become dysregulated and, if left unchecked, can lead to secondary injury pathologies.^41^ Excessive or persistent inflammation within the joint can lead to abnormal fibroblast overexpression of extracellular matrix (ECM) and trigger arthrofibrosis, on the one hand, and remodeling of the bone-cartilage unit and early PTOA on the other (Fig. 2).^29,36,41,56,57^ Inflammation also leads to pain and higher levels of substance P, a known pain sensitizer and activator of mast cells and fibroblasts that creates a positive immune feedback loop^29^ (Fig. 2). Improving the inflammatory balance during these early pro-fibrotic and chondro-dysfunction events is key to optimal healing and reducing pain (Fig. 2).

Restoring the inflammatory balance after ACL injury may be possible by controlling early proinflammatory cascades and the key genes and signaling pathways that drive secondary injury^41^ (Fig. 2). Key inflammatory inhibitors include inhibitors or antagonists of toll receptor (TLR), NF-κB, TNF-α, type I interferons (IFN-α and -β), type II IFN-γ, IL-1β, TGF-β1, suppressor of cytokine signaling proteins (e.g., SOCS), and inhibitors of the inflammasome (e.g., inhibitors of caspase-1, IL-1β, and IL-18).^58,59^ The signature genes underpinning fibrosis and cartilage remodeling are usually divided into two groups: anabolic genes for building ECM constituents and crosslinks (e.g., collagen type II, aggrecan, and fibronectin) and catabolic genes for degrading ECM constituents (e.g., MMP-1, MMP-3, MMP-13, ADAMTS-4, and ADAMTS-5) (Fig. 2).^37^ Reducing collagen type II, aggrecan, and fibronectin (Fn) gene expression may reduce cartilage anabolism.^29^ Similarly, as Sieker and colleagues recently concluded, therapies that inhibit MMPs and ADAMTS gene expression may restore chondro-balance and ameliorate early PTOA.^60^ Genes involved in regulating mitochondrial metabolism, such as mtCO3 (cytochrome C integrity), amp-k (metabolic sensor), sirt-1 (metabolic regulator), and PGC-1α (redox regulator),^42,61–63^ have also been implicated in secondary injury progression following ACL injury and PTOA, in particular in chondrocytes (Fig. 2).^62^

### Effect of Trauma of Surgery

As the patient goes to the operating room and anesthesia is induced, trauma is and convalescence begins.

Francis D. Moore ^64^ p 291

Another area that has received little attention is the effect of the trauma of surgery on perpetuating secondary damage after ACLR surgery^65^ (Table I). Surgical stress begins immediately after anesthesia and following the first incision, and continues during surgery. We recently showed that a single laparotomy, with no further surgery, induced a proinflammatory phenotype involving neuroendocrine stress, cortical excitability, immune activation, metabolic changes, and coagulopathy in the first 3 days.^66^ Accompanying this switch in phenotype was a 140-fold increase in IL-1β expression in the gut and a 6-fold increase in brain.^66^ Moreover, in the brain, there were significant increases in M1 muscarinic (31-fold) and α-1A-adrenergic (39-fold) receptor expression and expression of metabolic genes.^66^ These early and persistent changes after a single incision illustrate that despite anesthesia, the brain is still “wide awake” to receive damage-associated molecular patterns and other damage signals originating during surgery. To our knowledge, no study has investigated the effect of surgical trauma on the central nervous system and control of persistent inflammation and synovial and cartilage stress following ACLR surgery and the implications this may have on healing processes.

### Current ACL Interventions and Therapies are not Optimal

The burden of anterior cruciate ligament injuries and subsequent loss of readiness in these military warfighters highlights one of the most significant gaps in musculoskeletal injury care today.

Peebles and colleagues ^15^ p e12

Treatment options for ACL injury include nonoperative conservative management, arthroscopic versus open surgery and intra- versus extra-articular reconstruction.^2–4^ For more severe tears and ACL ruptures, arthroscopic reconstruction has become the standard-of-care.^2–4^ Ongoing controversial issues include graft selection, fixation, and timing of surgery.^2^ Drug therapies to reduce joint inflammation and pain have also met with limited success.^15,56,57,67,68^ Nonsteroidal anti-inflammatory drugs (NSAIDs), for example, can lead to gastrointestinal toxicity and bleeding,^68,69^ and opioids can lead to nausea, sedation, constipation, vomiting, and respiratory depression.^67,69^ Although the more commonly used NSAID Celecoxib is suitable for short-term pain relief, there is some evidence that it may impair soft tissue healing and tendon-to-bone healing and reduce mechanical stability of joints.^68^ Further clinical trials are required to investigate these possible negative effects of Celecoxib after ACLR surgery.^68^

### Future Directions in Joint Protection: Toward a System-based Approach

Achieving FDA approval for only one-in-ten drug indications that enter the clinic is a concerning statistic for drug developers, regulators, investors and patients.

Hay and colleagues ^70^

Currently there is no effective drug therapy that creates a “permissive environment” to prevent synovial and cartilage stress and reduce secondary complications. We argue that the lack of progress in this area is related to present day treat-as-you-go approach,^42,65,66^ which can lead to what U.S. surgeon William C. Shoemaker called: “an uncoordinated and sometimes contradictory therapeutic outcome.”^71^ This mindset appears to be a by-product of highly reductionist thinking. Although reductionism is essential for breaking complex systems down to its constituent parts for study, it does not do away with the system.^65,66^ Such an approach ignores the complexity of the system and may explain why there are so many failed clinical trials and why over 90% of new drugs fail to translate to humans.^70^ Failure to translate may also include poorly designed trials and the use of specific-pathogen specific experimental animals that fail to represent the “normal” microbiome physiology of the human undergoing surgery.^72,73^

The challenge for the future is to develop new “upstream” or “top down” system-based drug therapies that target the early stages of inflammation and immune dysfunction. We have been developing an adenosine, lidocaine, and magnesium (ALM) therapy for traumatic injury and hemorrhage,^74–77^ and more recently for total knee replacement (TKR) and ACL reconstruction.^78^ After showing short-term exposure of ALM solution was safe to human chondrocyte monolayers and improves cell viability,^79^ we examined the drug therapy in a rat model of TKR.^78^ We found that intra-articular ALM therapy significantly decreased systemic inflammation (IL-1β and IL-10), reduced fibrosis (**↓**TGF-β1, α-SMA, FGF1, PDGFA), and improved range of motion by 2-fold compared to saline controls over the 28-day study period.^78^ We further showed that ALM therapy reduced inflammatory NF-κB gene expression by 66% and MMP-13 gene expression by 50% in capsular tissue at day 28, with differences visualized histologically.^78^ We are currently evaluating the intravenous and intra-articular ALM therapy to reduce inflammation and expedite healing in males and females following ACL rupture and surgical reconstruction.

## CONCLUSIONS

ACL injuries are a major concern to military and civilian healthcare systems, with growth estimates of 4-6% per year, particularly among the young (15 to 25 years). Military personnel have a 10-fold higher incidence of ACL injuries than the general population, and woman from both populations are 2 to 8 times more likely to experience ACL injuries than men. Despite successful stabilization of the injured ACL, patient outcomes are variable with multiple injury phenotypes and risk stratification patterns for longer-term complications. Currently, there are no therapies that create a permissive healing environment to improve outcomes following ACLR surgery. It is our hypothesis that reducing early joint inflammation, immune dysfunction, and trauma-induced synovial stress may prevent secondary injury progression. We are developing an upfront system-based drug therapy to treat early inflammation and immune dysfunction and reduce the trauma of ACLR surgery with the goal to switch the injury phenotype into a healing phenotype with reduced arthrofibrosis and early PTOA.
